# An open label randomized Phase III trial of nivolumab or nivolumab plus ipilimumab vs platinum doublet chemotherapy (PT-DC) in patients with chemotherapy-naïve stage IV or recurrent non-small cell lung cancer (NSCLC) (CheckMate 227)

**DOI:** 10.1186/2051-1426-3-S2-P154

**Published:** 2015-11-04

**Authors:** Matthew Hellmann, Suresh Ramalingam, Martin Reck, Ken O'Byrne, Luis Paz-Ares, Christopher T Harbison, Prabhu Bhagavatheeswaran, Faith Nathan, Julie Brahmer

**Affiliations:** 1 Memorial Sloan Kettering Cancer Center, New York, NY, USA; 2 Winship Cancer Institute, Emory University, Atlanta, GA, USA; 3 Krankenhaus Grosshansdorf Gmbh, Ahrensburg, Germany; 4 Cancer Services, Princess Alexandra Hospital, Brisbane, QLD, Australia; 5 Hospital Universitario Virgen del Rocio, Sevilla, Spain; 6 Bristol-Myers Squibb, Princeton, NJ, USA; 7 Sidney Kimmel Comprehensive Cancer Center at Johns Hopkins, Baltimore, MD, USA

## Background

Patients with advanced NSCLC are treated with first-line PT-DC, which is associated with a median OS of 8–10 months and 1-year and 2-year survival rates of 30–40% and 10–15%, respectively. Nivolumab (a fully human IgG4 anti-programmed death-1 immune checkpoint inhibitor antibody) alone and in combination with ipilimumab (a fully human IgG4 cytotoxic T-lymphocyte antigen-4 immune checkpoint inhibitor antibody) has demonstrated encouraging clinical benefit across multiple tumor types. Two randomized Phase III trials demonstrated superior survival with nivolumab vs docetaxel in previously-treated patients with advanced squamous (SQ) (CheckMate 017) and non-squamous (non-SQ) NSCLC (CheckMate 057). Preliminary results of a Phase I study (CheckMate 012) of nivolumab with or without ipilimumab demonstrate acceptable safety and encouraging activity in first-line metastatic NSCLC across histologies. This Phase III trial (CheckMate 227) evaluates nivolumab monotherapy and nivolumab plus ipilimumab combination regimens vs PT-DC in patients with chemotherapy-naïve stage IV or recurrent SQ and non-SQ NSCLC.

## Methods

Adult patients with stage IV or recurrent NSCLC, ECOG performance status ≤1, no prior systemic anti-cancer therapy, and measureable disease per RECIST version 1.1 are eligible. Tissue will be evaluated for programmed death-ligand 1 (PD-L1) expression during screening. Patients who have untreated CNS metastases are ineligible. Patients will be randomized to nivolumab monotherapy, nivolumab plus ipilimumab combination regimens, or PT-DC. PT-DC will be administered according to histology (gemcitabine with cisplatin or carboplatin for SQ and pemetrexed with cisplatin or carboplatin for non-SQ). Patients will receive treatment until progression or unacceptable toxicity. The co-primary endpoints are overall survival and progression-free survival in patients receiving nivolumab monotherapy or nivolumab plus ipilimumab combination regimens vs patients receiving PT-DC. Secondary endpoints include objective response rate in nivolumab monotherapy or nivolumab plus ipilimumab combination regimens vs PT-DC and disease related symptom improvement measured by the Lung Cancer Symptom Scale in all patients.

## Trial registration

ClinicalTrials.gov identifier NCT02477826.

